# Mixed Germ Cell Tumor with Extensive Yolk Sac Tumor Elements in the Frontal Lobe of an Adult

**DOI:** 10.1155/2012/473790

**Published:** 2012-12-24

**Authors:** Toshihide Takahashi, Eiichi Ishikawa, Yosuke Masuda, Tetsuya Yamamoto, Taiki Sato, Makoto Shibuya, Akira Matsumura

**Affiliations:** ^1^Department of Neurosurgery, Faculty of Medicine, University of Tsukuba, Tsukuba, Ibaraki, 305-8575, Japan; ^2^Department of Pathology, Faculty of Medicine, University of Tsukuba, Tsukuba, Ibaraki, 305-8575, Japan; ^3^Department of Pathology, Tokyo Medical University Ibaraki Medical Center, Ami, Ibaraki 300-0395, Japan

## Abstract

Intracranial nongerminomatous germ cell tumors (NGGCTs) in unusual locations are extremely rare. Here, we report a case of a yolk sac tumor in the frontal lobe in a middle-aged patient. A 42-year-old man was admitted to our hospital for headache and nausea. Magnetic resonance imaging (MRI) showed an enhanced mass lesion with a marked cyst component. The serum alpha-fetoprotein (**α**FP) level was extremely high. Histological examination of specimens after subtotal removal revealed a primary mixed germ cell tumor with extensive yolk sac tumor elements, often referred to as an intracranial “yolk sac tumor.” The preoperative diagnosis of NGGCTs in unusual age and locations is extremely difficult. Clinicians should consider the possibility of NGGCTs, including yolk sac tumors, when intracranial tumors with unusual MRI findings are encountered.

## 1. Introduction

Nongerminomatous germ cell tumors (NGGCTs) of the central nervous system (CNS) are rare, accounting for fewer than 1% of primary brain tumors, and they occur predominantly in children or young adults [1, 2]. Twelve percent of NGGCTs are mixed germ cell tumors with yolk sac tumor elements, and 2% are pure yolk sac tumors [3]. Most NGGCTs, including yolk sac tumors, are located in pineal lesions or suprasellar lesions, and they rarely originate from basal ganglia and other lesions [3]. Here, we report a case of a yolk sac tumor in the frontal lobe in a middle-aged patient. 

## 2. Case Report

**Figure 1:**
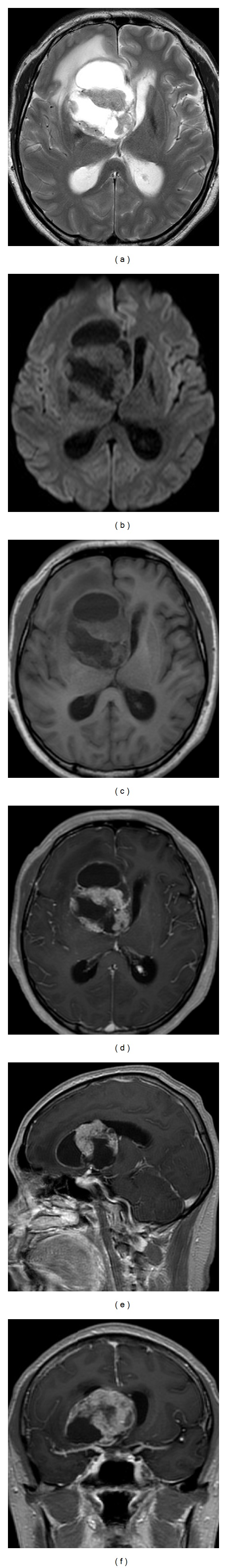
Transaxial ((a)–(d)), sagittal (e), and coronal (f) magnetic resonance imaging (MRI) views before surgery showing a huge lesion (58 mm maximum diameter) with marked cyst components. This lesion extends from the right caudate nucleus to the anterior of the lentiform nucleus and the white matter of the frontal lobe. A T2-weighted image (WI) shows the mass as a hyperintense lesion with slight edema around the lesion (a). The right lateral ventricle and third ventricle are pushed to the upper left side, and the inferior horns of both lateral ventricles are mildly enlarged with paraventricular hyperintensity. The tumor exhibits isointensity in a diffusion-WI and a T1WI ((b), (c)). T1WIs with gadolinium (Gd) show a well-demarcated enhanced mass ((d)–(f)).

**Figure 2:**
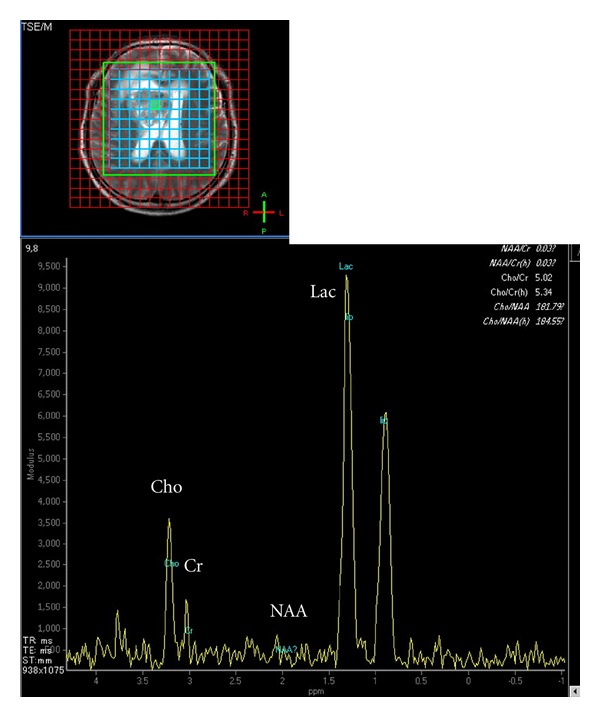
Proton magnetic resonance spectroscopy (1H-MRS) analysis of a voxel of interest in the nodule (a green area in upper figure) showing an increased ratio (5.02) of choline (Cho) to creatine (Cr) (Cho/Cr) and an apparent peak for lactate (Lac), which is correlated highly with tumor malignancy (lower figure). The peak for N-acetylaspartate (NAA) is within the background noise level.

A 42-year-old Japanese man was admitted to our hospital for headache and nausea. These symptoms had progressed for 4 days before admission, and he was gradually unable to walk steadily. Magnetic resonance imaging (MRI) revealed a mass lesion with marked cyst components. The lesion had a maximum diameter of 58 mm (Figure 1). T1-weighted images (WIs) after gadolinium administration showed ring enhancement. T2WIs showed slight edema around the lesion. Proton magnetic resonance spectroscopy (1H-MRS) analysis of the nodule showed an increased ratio of choline to creatine and an apparent peak for lactate, which is strongly correlated with tumor malignancy. In contrast, the peak for N-acetylaspartate (NAA) was within the background noise level (Figure 2). The neuroimaging diagnosis was a primary malignant brain tumor such as glioblastoma multiforme (GBM) or a metastatic brain tumor. The patient underwent subtotal removal of the lesion via a fronto-temporal craniotomy. 

**Figure 3:**
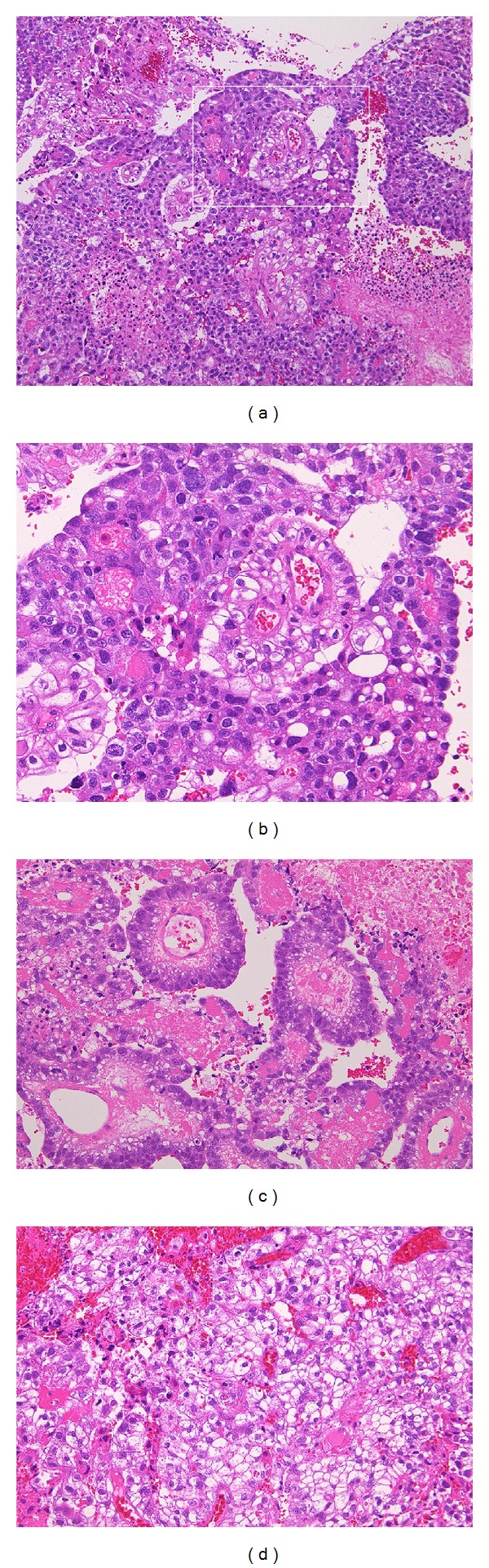
Hematoxylin and eosin stain showing papillary structures with a broad necrotic focus, mitosis, and Schiller-Duval bodies ((a): ×40, (b): ×200). Typical Schiller-Duval bodies ((c): ×100) and relatively loose area with microcystic component ((d): ×100) are shown.

**Figure 4:**
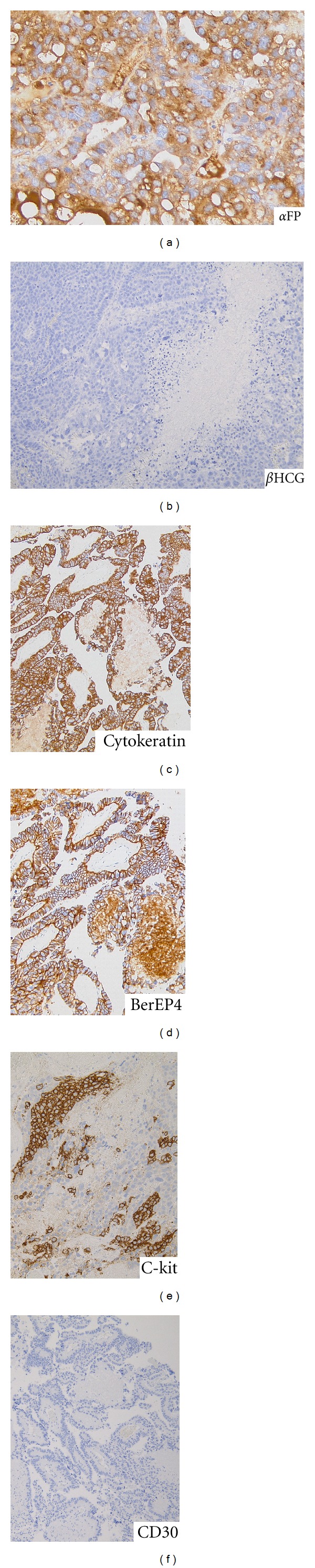
Immunohistochemical staining shows that majority of the tumor cells are strongly positive for alpha-fetoprotein (*α*FP) ((a): ×400) and negative for beta subunit of human chorionic gonadotropin (*β*HCG) ((b): ×100). They are strongly positive for wide-spectrum cytokeratin ((c): ×100), BerEP4 ((d): ×100), partially positive for CD117 (c-kit) ((e): ×200), and negative for CD30 ((f): ×100).

**Figure 5:**
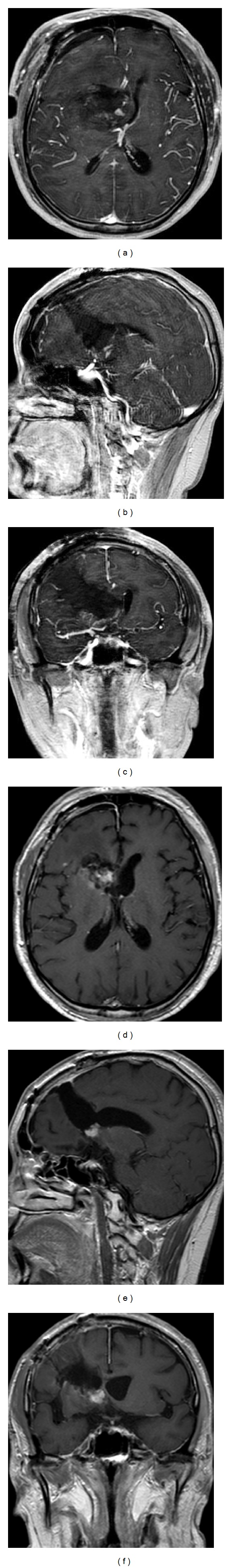
MRI after surgery showing that the mass lesion has been subtotally removed. T1WIs with Gd just after surgery show that more than 95% of the enhanced mass is enucleated and that a small residual tumor remains near the third ventricle and the caudate nucleus ((a)–(c)). T1WIs with Gd after 2 cycles of chemotherapy show a small residual tumor located in the same region, which has decreased in size after transient expansion after the 1st cycle of chemotherapy ((d)–(f)).

**Figure 6:**
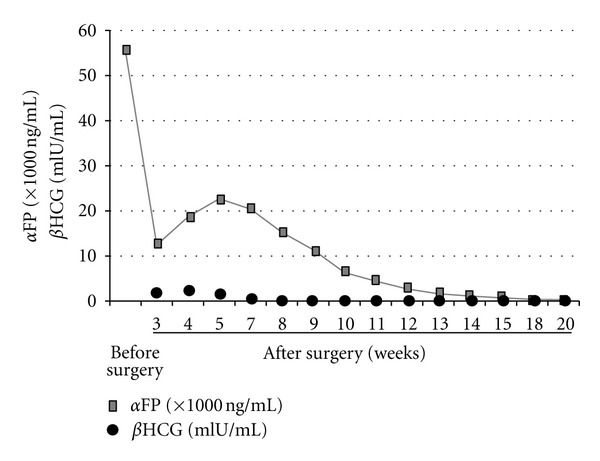
Serum concentrations of *α*FP (●) and *β*HCG (■) in the patient. The *α*FP concentration is extremely high (55500 ng/mL) before surgery. The concentration of *α*FP decreases after the surgery and subsequent chemoradiotherapy.

Histological examination of the surgical specimens revealed papillary structures with Schiller-Duval bodies (Figure 3). The tumor cells were strongly positive for wide-spectrum cytokeratin, BerEP4, and alpha-fetoprotein (*α*FP); partially positive for CD117 (C-kit); negative for glial fibrillary acidic protein (GFAP), S-100 protein, CD30, and the beta subunit of human chorionic gonadotropin (*β*HCG) (Figure 4). These results indicated that the majority of the specimens were yolk sac tumor elements, although these specimens also contained an extreme minority of germinoma component. These results were consistent with the high level of serum *α*FP (55500 ng/mL) observed before surgery, which was confirmed after surgery. A testicular ultrasound and a computed tomography (CT) scan of the chest and abdomen showed no lesions outside of the brain, and the final diagnosis was a primary mixed germ cell tumor of the CNS with extensive yolk sac tumor elements, often referred to as a “yolk sac tumor.” The patient underwent 5 courses of chemotherapy with 900 mg/m^2^ of ifosfamide, 20 mg/m^2^ of cisplatin, and 60 mg/m^2^ of etoposide along with 30.6 Gy of radiotherapy to the whole brain and a local boost of 30.6 Gy. MRI after treatment showed a reduction in the size of the lesion (Figure 5). The serum level of *α*FP also decreased after treatment (Figure 6). The patient remained neurologically intact with mild headache (90% of Karnovsky performance status (KPS)) and *α*FP level returned to within normal range 12 months after the surgery. 

## 3. Discussion

The authors retrospectively speculate that the tumor in the present case originated from the basal ganglia (most likely from the caudate nucleus) and extended into the frontal lobe. Detailed descriptions of intracranial yolk sac tumors in the frontal lobe or the basal ganglia have been published for only 7 cases, including our case [4–10]. In all of the previous cases, the tumors developed in patients no older than 18 years; the present case is the first report of a yolk sac tumor in this location in a middle-aged patient. 

In the present case, the preoperative diagnosis by neuroradiologists was GBM or metastatic brain tumor. However, retrospectively, various MRI findings including the existence of an enhanced nodule with cystic components lacking severe peritumoral edema and the very low peak for NAA on 1H-MRS were unusual for GBM. Similarly, in the previous cases of primary intracranial yolk sac tumor in unusual locations, the preoperative diagnosis was difficult [9]. The authors speculate that the serum *α*FP level will be the most useful marker for preoperative diagnosis. However, this value was measured before surgery in only 5 cases (23%) out of the previous cases of CNS yolk sac tumors located in unusual areas [5, 6, 10–12]. In our case, the preoperative serum *α*FP level, which was evaluated for the differential diagnosis of metastatic brain tumors along with other tumor markers, was extremely high, and the *α*FP level decreased after surgery. 

In conclusion, the preoperative diagnosis of NGGCTs in unusual age and locations is extremely difficult. Clinicians should consider the possibility of NGGCTs, including yolk sac tumors, when patients present with intracranial tumors with unusual MRI findings. 

## Abbreviation

(*α*FP): Alpha-fetoprotein
(*β*HCG): Beta subunit of human chorionic gonadotropin
(Cho): Choline
(CNS): Central nervous system
(Cr): Creatine
(Gd): Gadolinium injection
(GBM): Glioblastoma multiforme
(GFAP): Glial fibrillary acidic protein
(KPS): Karnovsky performance status
(Lac): Lactate
(MRI): Magnetic resonance imaging
(NAA): N-acetylaspartate
(NGGCTs): Nongerminomatous germ cell tumors
(1H-MRS): Proton magnetic resonance spectroscopy
(WIs): Weighted images.

## References

[1] Dolecek TA, Propp JM, Stroup NE, Kruchko C (2012). CBTRUS statistical report: primary brain and central nervous system tumors diagnosed in the United States in 2005–2009. *Neuro-Oncology*.

[2] The Committee of Brain Tumor Registry of Japan (2009). Report of brain tumor registry of Japan (1984–2000) 12th edition. *Neurologia Medico-Chirurfica*.

[3] Matsutani M, Sano K, Takakura K (1997). Primary intracranial germ cell tumors: a clinical analysis of 153 histologically verified cases. *Journal of Neurosurgery*.

[4] Honda M, Baba H, Yonekura M, Iseki M (2005). Cerebral composite atypical teratoid/rhabdoid tumor and yolk sac tumor in the frontal lobe of an infant: case report. *Neurologia Medico-Chirurgica*.

[5] Masuzawa T, Shimabukuro H, Nakahara N, Iwasa H, Sato F (1986). Germ cell tumors (germinoma and yolk sac tumor) in unusual sites in the brain. *Clinical Neuropathology*.

[6] Netalkar AS, Sharma RR, Mahapatra AK (2001). An unusual presentation of an intra-parenchymatous frontal yolk sac tumour: case report. *Neurology India*.

[7] Oshita N, Yamashita K, Gotou K, Nagata I, Ueda H, Mitani T (1993). A case of endodermal sinus tumor in the basal ganglia associated with Down’s syndrome. *No Shinkei Geka*.

[8] Sugawara T, Tsurumi Y, Kuwahara K, Katakura R, Suzuki J (1984). Primary intracranial yolk sac tumor developing in the frontal lobe from the inside of the sphenoidal ridge. *No Shinkei Geka*.

[9] Tsugu H, Oshiro S, Ueno Y (2009). Primary yolk sac tumor within the lateral ventricle—case report. *Neurologia Medico-Chirurgica*.

[10] Wang CH, Hsu TR, Yang TY (2010). Primary yolk sac tumor of bilateral basal ganglia. *Journal of the Chinese Medical Association*.

[11] Abdennebi B, Bendisari K, Mansouri B, Abada M (1988). Intracranial tumor of vitelline sac origin. A case report. *Neurochirurgie*.

[12] Wada H, Kubo M, Wada T (1995). A case report of a 6-year-old boy with intracranial yolk sac tumor treated by VAB-6 regimen. *No Shinkei Geka*.

